# Development of a web software prototype to support retirement planning *

**DOI:** 10.1590/1518-8345.3024.3169

**Published:** 2019-08-19

**Authors:** Paloma de Souza Cavalcante Pissinati, Yolanda Dora Martinez Évora, Mariana Angela Rossaneis, Raquel Gvozd, Marcio Souza dos Santos, Maria do Carmo Fernandez Lourenço Haddad

**Affiliations:** 1 Universidade Estadual de Londrina, Londrina, PR, Brasil.; 2 Prefeitura Municipal de Rolândia, Secretaria Municipal de Saúde, Rolândia, PR, Brasil.; 3 Universidade de São Paulo, Escola de Enfermagem de Ribeirão Preto, Centro Colaborador da OPAS/OMS para o Desenvolvimento da Pesquisa em Enfermagem, Ribeirão Preto, SP, Brasil.; 4 Universidade Norte do Paraná, Londrina, PR, Brasil.

**Keywords:** Medical Informatics, Information Technology, Software, Software Engineering, Healthy Aging, Retirement, Informática Médica, Tecnologia da Informação, Software, Engenharia de *Software*, Envelhecimento Saudável, Aposentadoria, Informática Médica, Tecnología de la Información, Programas Informáticos, Ingeniería de Programas Informáticos, Envejecimiento Saludable, Jubilatión

## Abstract

**Objective:**

To develop a web software prototype to support retirement planning.

**Method:**

This is a methodological research, applied and based on the principles of prototyping model, which followed the steps of communication, planning, prototype creation, functional tests and consolidation of web software version 1.

**Results:**

The functions of the web software prototype were defined from a flowchart and scope. In the creation stage, the screens that integrated the prototype, composed by interview, were projected from the filling of the Retirement Resources Inventory, screen of access to support planning materials, including lectures, scientific texts, and technical materials, retirement news screen, experiences screen, which allow users to post retirement expectations and comment on other users’ posts. After performing tests, the prototype was made available at www.aposentarsecomsaude.com.br *.*

**Conclusion:**

the web software prototype consists of an interactive environment in which the user feels active in the reflection process about the retirement along the different screens. With clear language and expressions that are easily understood by the public, they are applicable to users of different professional profiles.

## Abstract (pt)

**Objetivo:**

desenvolver um protótipo de *web software* de apoio ao planejamento da aposentadoria.

**Método:**

trata-se de uma pesquisa metodológica, aplicada, pautada nos princípios do modelo de prototipação, que seguiu as etapas de comunicação, planejamento, criação do protótipo, testes de funcionamento e consolidação da versão 1 do *web software* .

**Resultados:**

as funções do protótipo do *web software* foram definidas a partir de um fluxograma e do escopo. Na etapa de criação foram projetadas as telas que integraram o protótipo, composto por entrevista, a partir do preenchimento do Inventário de Recursos para a Aposentadoria, tela de acesso aos materiais de apoio ao planejamento, incluindo palestras, textos científicos e materiais técnicos, tela de notícias sobre a aposentadoria, tela de vivências, as quais permitem aos usuários publicar expectativas em relação à aposentadoria e comentar publicações de outros usuários. Após a realização dos testes de funcionamento, o protótipo foi disponibilizado no endereço www.aposentarsecomsaude.com.br.

**Conclusão:**

o protótipo do *web software* consiste em um ambiente interativo, no qual o usuário sente-se ativo no processo de reflexão sobre a aposentadoria ao longo das diferentes telas. Com linguagem e expressões claras, de fácil entendimento ao público distinto a que se destina, torna-se aplicável aos usuários de diferentes perfis profissionais.

## Abstract (es)

**Objetivo:**

desarrollar un prototipo de *web software* de apoyo a la planificación de la jubilación.

**Método:**

se trata de una investigación metodológica, aplicada, pautada en los principios del modelo prototipado, que siguió las etapas de comunicación, planificación, creación del prototipo, pruebas de funcionamiento y consolidación de la versión 1 del *web software* .

**Resultados:**

las funciones del prototipo del web software se definieron a partir de un diagrama de flujo y del ámbito. En la etapa de creación fueron proyectadas las pantallas que integraron el prototipo, compuesto por entrevista, completando el Inventario de Recursos para la Jubilación, pantalla de acceso a materiales de apoyo a la planificación, incluyendo conferencias, textos científicos y materiales técnicos; pantalla de noticias sobre la jubilación, pantalla de vivencias, las cuales permiten a los usuarios publicar expectativas en relación a la jubilación y comentar publicaciones de otros usuarios. Después de la realización de las pruebas de funcionamiento, el prototipo estuvo disponible en la dirección www.aposentarsecomsaude.com.br.

**Conclusión:**

el prototipo del *web software* consiste en un ambiente interactivo, en el cual el usuario se siente activo en el proceso de reflexión sobre la jubilación a lo largo de las diferentes pantallas. Con lenguaje y expresiones claras, de fácil entendimiento al público distinto al que se destinan, se vuelve aplicable a los usuarios de diferentes perfiles profesionales.

## Introduction

In recent years, Brazil has been faced with a significant intensification of the aging process associated with a social security crisis^1^ , which contributes to a scenario of insecurity not only for the elderly, but also for those who are still far from retirement. In this sense, it is crucial that the worker becomes aware as soon as possible about the importance of adopting planning strategies that lead to a healthy shutdown^2^ .

The decision to retire can be a difficult task for some workers, who tend to take years to start thinking about it. Faced with the possibility of becoming an obstacle, the individual needs to be stimulated to initiate planning actions as a strategy to facilitate adaptation to the new life span^3^ .

A cohort study conducted in the United States of America, with more than 37,000 people, aged over 50, showed that successful retirement was a process of multiple influences, involving resources and behaviors developed through actions of planning. These actions include the need to prepare for economic, social, family, physical, psychological and cognitive aspects, as well as to adopt beneficial health behaviors, financial care, and the use of support programs^2^ .

Among the instruments that contribute to the reflection of the worker on retirement planning, the Retirement Resources Inventory (RRI)^4^ , translated and validated in Brazil^5^ stands out. These instruments, such as the RRI, become important tools of action for Retirement Preparation Programs (RPPs), which help workers reflecting on the changes they may experience after leaving work^6^ .

However, maintaining active RPPs and promoting employee participation in face-to-face meetings is a challenge today, mainly due to the shortage of qualified human resources to conduct the meetings and the intensity of individuals’ daily activities.

In this sense, the software development becomes an alternative to complement actions and promote reflection on retirement planning, even when the worker is not available to attend face-to-face meetings. Software is understood to be a flexible, easily accessible, versatile and robust technological resource that can be used by numerous people in the same period of time, regardless of the locality^7^ .

Among the different types of software, the web software stands out, also known as web applications, which is characterized by being hosted on a server with remote and secure access to the users through a web browser, having the internet as environment of development and execution. Web software allows to perform functions of greater complexity, when compared to web sites since, besides providing information, they include resources - tools, calculations and feedback functions - in real time, interactively, through exchanges with the user, besides providing bank data storage and analysis, and eliminating the need to install a hardware in the computer^8^ .

In the area of health and nursing, software development allows integrating actions, directing the work process, and contributing to the management of services. However, many software systems are aimed at diagnosis of diseases, nursing care, and teaching. Therefore, there is a gap in studies that apply them in other ways, given the diverse possibilities of using these systems^9 - 11^ , such as the ability to support the retirement planning process.

The objective of this study was to develop a web software prototype to support retirement planning.

## Method

This is a methodological study of the applied type. The development of the web software prototype adopted as a methodological reference the steps of the prototyping model^12^ , namely: communication, planning, prototype creation, evaluation, functional tests, and consolidation of version 1 of web software.

For the development of the technical aspects of the web software prototype, the authors hired a company specialized in web development and the developers had training in systems development and graphic designer with specialization in web development. The process of planning the operation, selection of contents and presentation of the screens was carried out by the authors themselves, who had training in nursing and experience in retirement planning.

In the planning stage, the web software prototype was developed, through which the aspects visible to the users were established, such as the layout and presentation of the screens^12^ . Also, the flowchart and the scope with the functions of the prototype were set.

A sociodemographic and occupational characterization questionnaire was also developed to be answered by the user when accessing the interview page, which included the variables age, sex, marital status, number of children, monthly family and individual income, profession, whether the user was employed at the time that he/she accessed the web software, work shift, whether he/she worked overtime, whether he/she had another concomitant work, whether he/she was studying at the moment, which course he/she was doing, what the retirement forecast was, among others.

In addition, the RRI, translated and validated in Brazil^4^ , was computerized and inserted in the web software prototype. The purpose of this Instrument is to gather resources relevant to the achievement of well-being during retirement. It is organized in six domains, encompassing physical, financial, social, emotional, cognitive, and motivational aspects, reinforcing the premise that promoting a healthy retirement requires multidimensional planning.

The Inventory is composed of 30 items, scored using a five-point Likert scale, distributed in four resources types (RT): RT1, which comprises the physical aspects; RT2, the financial aspects; RT3, the social aspects and RT4, the emotional, cognitive, and motivational aspects.

From the completion of the questionnaire, after reading and accepting the Informed Consent Form made available in digital format, users were directed to specific screens with support materials.

The materials to support retirement planning were selected from an initial bibliographical survey, conducted between December 2016 and August 2017. Electronic sites (Google^®^and Youtube^®^platform), scientific databases - Latin American and Caribbean Health Sciences Literature (LILACS); Medical Literature Analysis and Retrieval System Online (MEDLINE); *Biblioteca Virtual da Saúde* (BVS) and the Biblioteca Virtual da Saúde de Psicologia (BVSPIS) and the Public Medline search engine (PUBMED) were consulted, from the combination of the descriptors controlled by the *Descritores em Ciências da Saúde* (DECS): loss; retirement benefits; retirement; retired; feeling.

Video-recorded lectures and reports were also included and made available in free access, as well as scientific texts, published in Portuguese language and available in full, related to the planning of retirement and the resources needed to prepare the worker. After this selection, the contents subsidized the “Interview and News” screen of the web software prototype. Then, the contents started to be updated weekly in the news tab and whenever the authors detected this need in the other tabs.

Also, a space for posting brief news related to retirement was elaborated as a way to keep users up to date on the topic. Finally, the interactive environment of the web software prototype was structured so that users can post their experiences and expectations regarding retirement, and also exchange messages with other workers who access this screen. On this screen, in order to guarantee the confidentiality and ethics of personal information, the users had the option to choose whether they wanted to identify themselves and make their names visible to other users in the experiences tab, or they could choose to remain anonymous.

After completing the planning stage and defining how the instruments and materials would be inserted in the web software, the technical development of the system was started. A computerized sketch containing the main characteristics was elaborated, denominated prototype^12^ .

The development of the prototype was divided in three main stages: the design, which included the creation of the layout; the frontend, which included the definition of the user-visible interface, with the screens and the icons for accessing the Retirement Resources Inventory, support materials and access to the posted news; and the backend, which constituted the face not visible to the user, responsible for information processing, export and storage in the database.

For the creation of the layout of the web software prototype, the Adobe Photoshop^®^program was used, by selecting only free access images. For the construction of the frontend and the backend, the developers used the Sublime Text^®^, which allowed them to save the files in the programming and markup format. In addition, the technical staff also used the Apache, My Structured Query Language (MySQL) and Hypertext Preprocessor (PHP-MAMP^®^) software systems as facilitators for the prototyping tests by simulating a web environment on the computer itself.

In the construction of the user interface (frontend), in order to guarantee a responsive and adaptable design for different formats of screens and browsers, we chose to use Cascading Style Sheets (CSS) markup languages, which allowed us to delineate the style fonts, spacing, and color of the web software. The HyperText Markup Language (HTML) was also used, which provided the structural aspects of the page, allowing the insertion of documents, videos, images and other file formats a well as JavaScript, which consists of a third layer of development, which ensures user interaction with the items inserted in the page.

As for the backend of the web software prototype, we chose to use the programming language in Hypertext Preprocessor (PHP) to process information entered by the user. The data processed was stored in a database in MySQL format, which uses Structured Query Language (SQL). combined with PHP for application (P4A), thus allowing the export of the data in the Excel^®^format for further analysis.

The management of the contents and data entered by the users was done through the access to the administrative screen, available at www.aposentarsecomsaude.com.br/admin. Upon accessing it, the researchers inserted and updated the contents of the web software prototype, and downloaded the database of the users’ answers to the RRI and the posts in the experiences screen.

To ensure the *management of access* to the web software, the Google Analytics^®^feature was used, which is a free web tool that works through HTML codes to track information related to access.

Thus, we adopted as indicators to monitor accesses of the web software prototype: the average pages/contents are visualized per day; the percentage of users who accessed the web software and left without accessing any page or internal content; the number of users who are accessing the web software prototype at that moment (online users); the time the user remains active in the prototype, including access to content, clicks, and all activities developed; the average session duration; the average number of pages accessed during the session; the percentage of users who accessed the prototype for the first time and of users who returned; the percentage of users who accessed it according to the type of device (computer, mobile phone or tablet) and the number of accesses that were made from the direct typing of the prototype link in the browser, as well as the accesses coming from the list of user favorites.

During the development of the prototype, tests were carried out to verify the functionality of the user interface, which included: testing of interface characteristics; testing of web environments; link testing and forms testing.

Once the functional corrections were made, version 1 of the web software was made available. The development of the prototype web software occurred between July 2016 and June 2018.

The study respected the ethical precepts, being approved by the Committee of Ethics in Research Involving Human Beings, according to Opinion no. 1,543,255.

## Results

The prototype was defined in its scope as a web software capable of collecting, storing and processing data referring to aspects of retirement well-being, as well as providing support materials for user planning.

**Figure 1:**
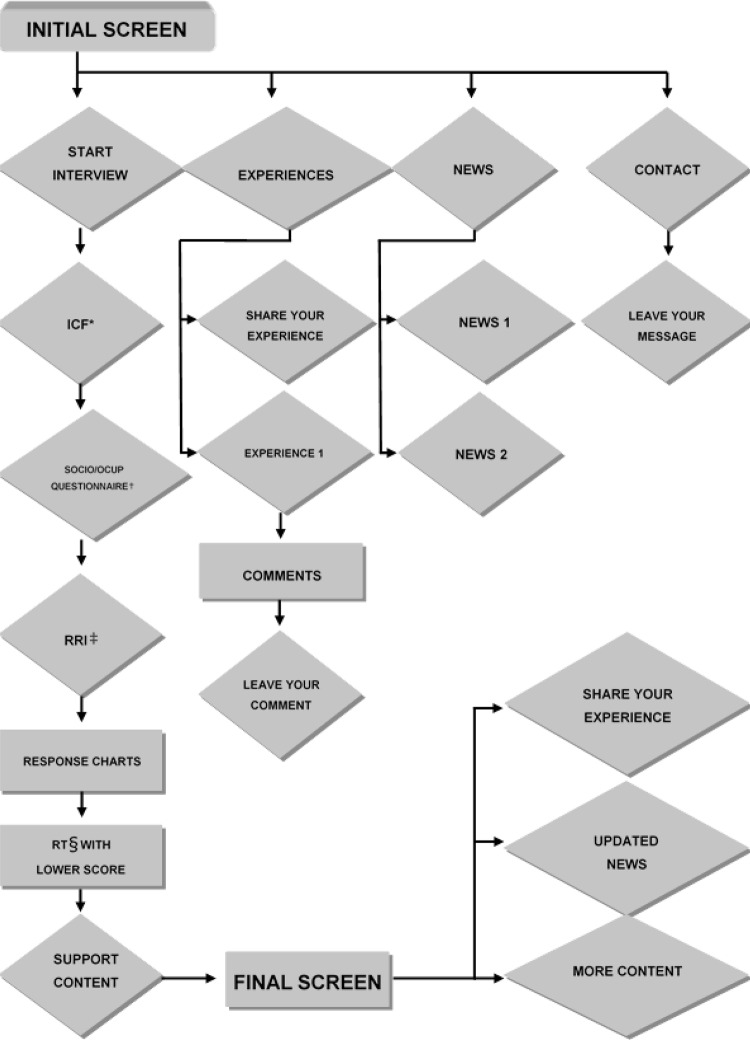
*ICF: Informed Consent Form; †SOCIO/OCCUP: Sociodemographic and Occupational; ‡RRI: Retirement Resources Inventory; §RT: Resource - Functional flowchart of the web prototype software to support retirement planning. Maringá, PR, Brazil, 2018

To begin the development of the web software prototype, it was necessary that the researchers elaborate the flowchart with all the screens that would integrate it, as well as its functions. The “ *Iniciar Entrevista* ” (“Start Interview”) icon was defined as the central element, through which the worker would respond to the sociodemographic and occupational questionnaire and the Retirement Resources Inventory, as shown in Figure 1 .

During the technical development of the web software prototype, the operation of the home screen and the presentation layout of each icon was defined. The aim was to ensure that all the functions were available to the users, with source in adequate size and an inviting environment.

**Figure 2:**
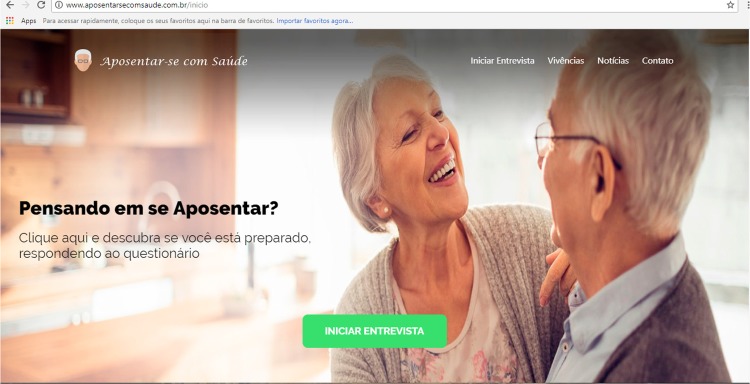
Web software prototype first screen to support retirement planning. Maringá, PR, Brazil, 2018

The central phrase “ *Pensando em se Aposentar? Clique aqui e descubra se está preparado, respondendo o questionário* ” (“Thinking of Retirement? Click here and find out whether you are prepared by answering the questionnaire”) was included to invite the user to respond to the Retirement Resources Inventory. In addition, icons were inserted in the upper right corner of the page to access the interview, news, experiences and contact with the developer team ( Figure 2 ).

When selecting the central action, Start Interview, the user is directed to a second screen with the Informed Consent Form and, after reading and agreeing, he is directed to the third screen, referring to the Sociodemographic and Occupational Characterization Questionnaire.

This screen was programmed for the user to fill it in using open and multiple choice questions. In order to ensure full completion, to export information to the Excel^®^database on the backend, all questions were designed with a symbol that made them mandatory, so that the user proceeded to the next screen only after completely responding to the questionnaire.

By going to the fourth screen, the user accessed the RRI questions. This inventory is divided into five sequential screens, with six multiple-choice questions, with mandatory completion.

After filling out the RRI, the user automatically received a copy of the responses in their e-mail along with the Informed Consent Form. In web software, he was directed to a fifth screen, in which he visualized a chart with the score obtained in the four dimensions of the questionnaire.

**Figure 3:**
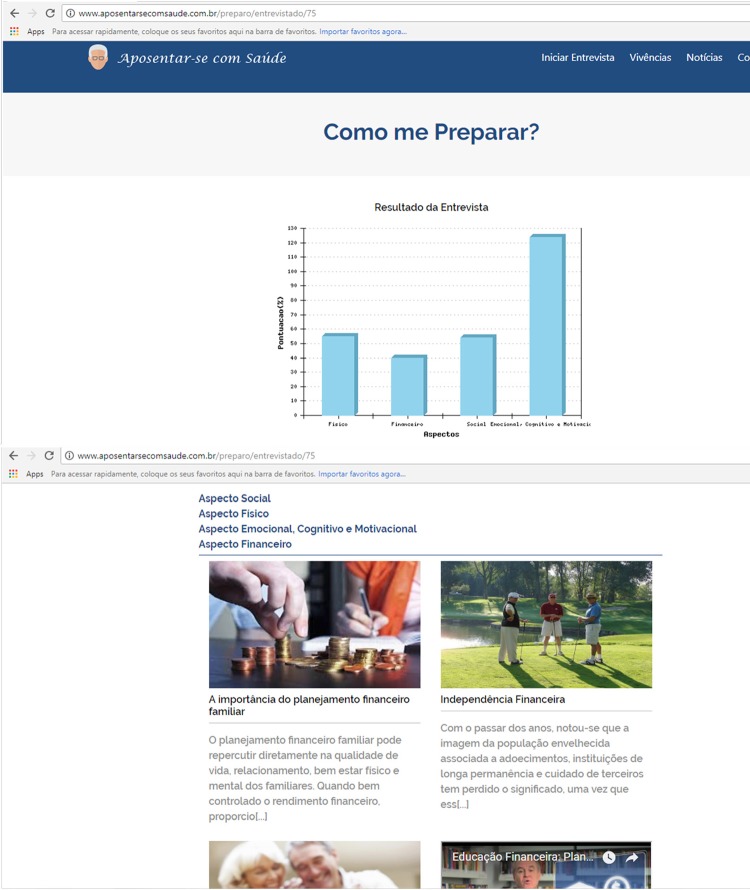
Final scoring screen and web software prototype support materials to support retirement planning. Maringá, PR, Brazil, 2018

Subsequently, specific support content was programmed, which included technical materials, scientific articles, videos made available on the network for free access and lectures related to the dimension in which the individual presented lower scores. The participant also had the option to access the materials for the other dimensions ( Figure 3 ).

In order to ensure that the user was able to return to the page with the final result of the questionnaire after browsing the contents, the questionnaire was made available in a new browser tab when clicking on the icons of each dimension. In this way, it was possible to return to the root of responses and explore materials from other dimensions.

**Figure 4:**
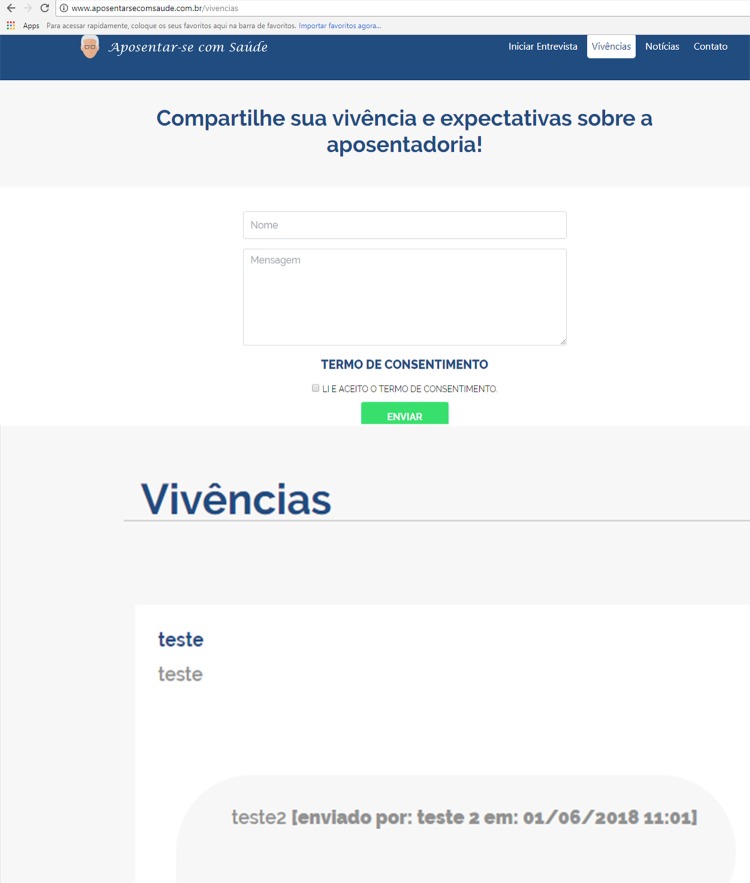
– *Vivências* (Experiences) screen of the web prototype software to support retirement planning. Maringá, PR, Brazil, 2018

The *Vivências* (Experiences) screen was also programmed, in which the user could post expectations, anxieties and share experiences in relation to retirement, as well as exchange messages with other people when inserting answers to previously published posts. It was sought to establish an interactive environment that resembled social networks, in which the individual had the possibility to comment and visualize the experiences ( Figure 4 ).

**Figure 5:**
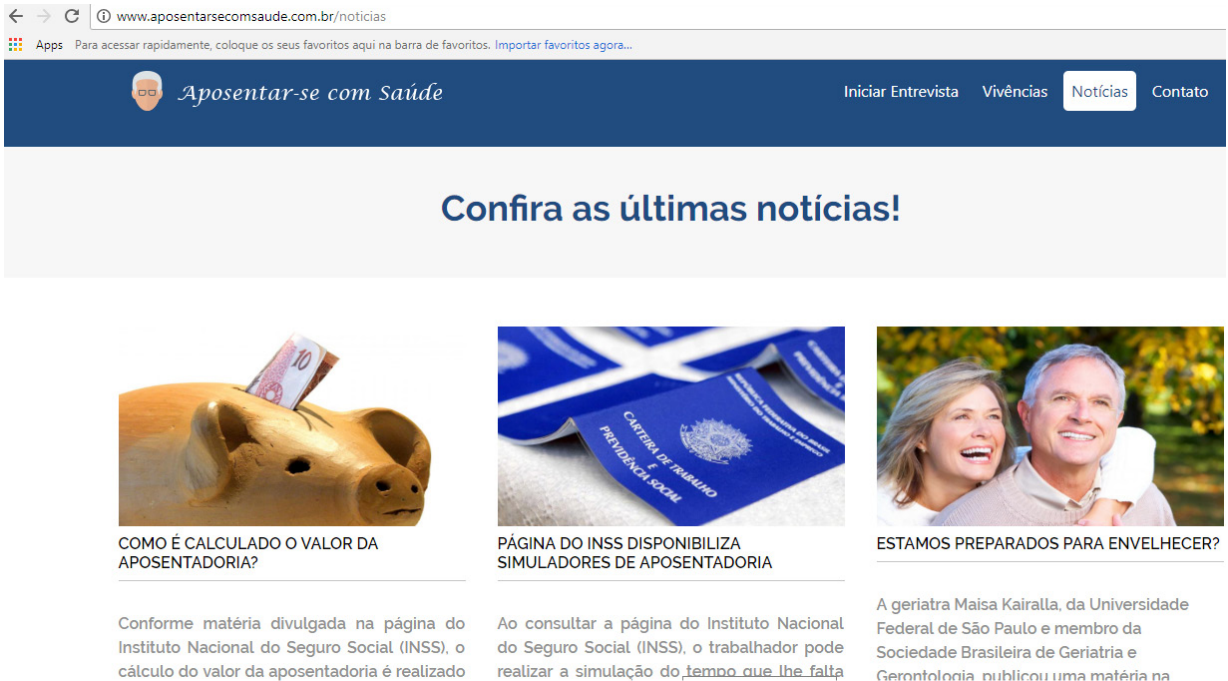
– *Notícias* (News) screen Web software prototype to support retirement planning. Maringá, PR, Brazil, 2018

The prototype also included a *Notícias* (News) screen, in which the user has access to updated contents weekly, related to the preparation for retirement, as shown in Figure 5 .

In order to increase interaction between researchers and users, the option *Contato* (Contact) was programmed, which allows sending messages directly to the management team of the web software prototype; another option made available was the e-mail “contato@aposentarsecomsaude.com.br “.

The development of the administrative area allowed the updating of contents directly by the team of researchers. It was also possible to view all users’ responses to the RRI, the posts and comments entered by the users in the Experience screen and to download all the data in an Excel^®^worksheet, in order to submit them to the analysis in later searches.

Functioning tests were carried out by the team of developers and researchers, continuously, throughout the creation of the prototype. We tried to detect possible errors in the aesthetics and visual content presented to the user; ensure proper functioning from different browsers, so that there is no visual distortion to the user; ensure that the user can access all content correctly by clicking the links available and also make sure that the questionnaires presented adequate size and data to avoid the loss of information inserted when clicking on icons, for example on the return function.

After correcting the malfunctions, version 1 of the web software Retiring Healthily was made available on the internet through the link www.aposentarsecomsaude.com.br, which was then monitored to evaluate users accesses. As a dissemination strategy and in order to expand access to the prototype, an access link was made available on the Portal of a Brazilian public university.

The management of access of the web software Retiring Healthily was carried out from the electronic platform Google Analytics^®^. According to the report, up to June 30, 2018, 337 users had accessed the prototype, of which 307 (91.10%) had accessed it for the first time and 30 (8.90%) had returned more than once.

The accesses came from different regions of Brazil, with 297 (88.13%) from the state of Paraná, in addition to accesses in Brasília, Rio de Janeiro, Bahia, Rio Grande do Sul and São Paulo. At the international level, there was access to the prototype in the city of Córdoba, Argentina.

A total of 1,563 page views were counted, corresponding to the number of contents accessed by users and users remained, on average, 4 minutes and 48 seconds in each session, with a total of 369 sessions.

The web software prototype presented a rejection rate of 34.15%, which represents access to a single page, without user interaction and permanence. An average of 4.24 pages were viewed during each session, excluding repeated access to the same page.

The web software prototype was developed in a way that allows modifications in all contents and screens in order to promote the continuous improvement of its functions and the insertion of new material to support the process of planning retirement. The markup and programming language was chosen to ensure that users could access the page from different digital platforms - through tablets, smartphones or computers - with the same image quality and execution of functions.

## Discussion

The development of a web software prototype to support retirement planning adopted as a central element the computerized version of the RRI so that, during its completion, users could identify the areas that presented greater weakness in relation to retirement planning^5^ . Thus, from the score displayed by means of a graph projected on the results screen, users accessed selected support materials, aimed to promote reflection on the aspect of lower punctuation, which corresponds to the greater weakness regarding the preparation for termination of work activities.

The elements that make up the ideal transition model for retirement include the choice and control over the right moment to leave work activities and, above all, access to resources that allow workers to prepare for that moment^13^ . The development of the web software prototype to support retirement planning represents one of the strategies to be used by the worker throughout their professional career, so as to allow the diagnosis of areas of greater weakness and raise reflection from support contents.

Still, some challenges for the development of web prototype software to support retirement planning are the ability to be attractive and to promote an interactive environment for end users. It is emphasized that, as a strategy for health promotion, the development of mobiles softwares technologies, which allow online interaction, has stood out in relation to the traditional media options^14^ .

The web software prototype is directed to a wide range of users with different sociodemographic and occupational characteristics. Therefore, technology should seek to integrate and make available content that is easy to understand and avoid excessive visual information to ensure agility in access and a standardized navigation flowchart^15^ .

The web prototype software was designed from a previously determined scope, with texts with accessible language and self-explanatory screens to those that access it. These strategies are consistent with the key aspects for the implementation of a successful health information technology, as described in a study carried out in the United States and the United Kingdom. These aspects include the importance of clarity about the objective to which it is intended and how much this technology will contribute to the solution of the problem. In addition, we can mention the search for balance in the writing and presentation of contents in the software screens so that they are understood by different users^16^ .

The web prototype software, besides supporting the worker in the planning the retirement, also allows the generation of a database, from the filling of interview and experiences. The use of this software by health professionals has several objectives, such as the collection and processing of information. The incorporation of such tools increases the possibility of analyzing epidemiological data with significant contributions to clinical decision making, patient quality and safety^17^ .

The functioning tests were performed with the purpose of correcting possible faults, ensuring the quality of the prototype and, also, obtaining the consolidation of version 1 and make it available in open access format. These procedures are the first steps in the evaluation of a new software, especially in the area of health, in which the aim is to ensure not only adequate performance, but also the safety of the information collected^18^ .

After having made available the version 1 of the web software Retiring Healthily, it was possible to verify the fast adherence of the users by the expressive amount of accesses performed in different regions of Brazil and the expressive number of page views in a short period of time. The use of an access manager contributes to knowing the usage profile, content preferences, completed forms, besides allowing to track users’ behaviors and thus monitor the quality and acceptability of the software^19^ .

Faced with the trend of technological incorporation in the health area, it is necessary for health professionals to develop skills to design, implement and monitor software systems in the work process. The involvement of health practitioners allows to align the management of technologies with the objectives of the institution^20^ .

It is highlighted, as a study’s potential, the establishment of a complementarity relationship between researchers and the technical team of developers. This feature allowed for continuous prototype’s enhancement as well as development in a short period of time and, above all, the active participation of the researching nurses in the conduction and structuring of all the screens and designer of the web software prototype.

The development of the web prototype software Retiring Healthily consists of an innovative intervention strategy in retirement planning. The ability to provide a diagnosis of the area in which the user presents greater fragility in relation to the preparation for the termination of work activities and the immediate access to support contents to the refection process increases the potential of contributions of the prototype, also associated to the possibility access from different devices - tablets, smartphones and computers.

In addition, the prototype may contribute to the clinical practice of nurses that work in the management of occupational age and health, since they may use the web prototype software as a work tool for the care and to map how the workers identify themselves in relation to the planning of retirement. From the adoption of the prototype, they can elaborate interventions specific to the needs of each individual, in order to stimulate the preparation and lead to a healthy retirement.

As a limitation of the study, there was the difficult to find contents that could be applied to users with different sociodemographic and occupational characteristics and to adapt them to an accessible language, so that the web prototype software could be adopted by institutions and individuals in the different professional areas. Therefore, the development of future studies is suggested in order to analyze the usability by workers and the contributions to the process of reflection on retirement.

## Conclusion

In this study, we aimed to develop a web prototype software to support retirement planning.

The scope and flowchart of the prototype advocated the development of an interactive environment in which the user had the possibility to feel as an active participant in the reflection about retirement throughout the different screens. In addition, attractive images were used to refer to the tranquility one can enjoy from a planned retirement, as well as the adoption of clear language and expressions, which are easily understood by the intended audience.

The adoption of such resources, associated to continuous testing to improve the functioning of the prototype, contributed to the adherence of users in the short term. Therefore, the insertion of the access link to the web prototype software Retiring Healthily on public and private labor institutions websites should be encouraged as a way to stimulate the worker to reflect on the planning of retirement. The free access to this technology will allow the wide incorporation in the most diverse scenarios and for different professional categories.
